# Health, Social and Recidivism Outcomes Among People Who Have Been Incarcerated in New South Wales, Australia: Study Protocol and Cohort Profile for the Prison Outcomes STudy (POST)

**DOI:** 10.1111/dar.70161

**Published:** 2026-06-21

**Authors:** Louisa Degenhardt, Michael Farrell, Michael Doyle, Jack Stone, Chrianna Bharat, Matt Hickman, Marianne Martinello, Don Weatherburn, Kimberlie Dean, Joe Coyte, Christel Macdonald, Mary Ellen Harrod, Luke Grant, Gloria Larman, Peter Vickerman, Colette McGrath, Peter Thompson, Alison Churchill, Greg Dore, Thomas Santo

**Affiliations:** ^1^ National Drug and Alcohol Research Centre UNSW Sydney Sydney Australia; ^2^ The University of Sydney Sydney Australia; ^3^ Population Health Sciences, Bristol Medical School University of Bristol Bristol UK; ^4^ Kirby Institute, UNSW Sydney Sydney Australia; ^5^ School of Psychiatry UNSW Sydney Sydney Australia; ^6^ The Glen Centre Chittaway Point Australia; ^7^ NSW Users and AIDS Association Sydney Australia; ^8^ Corrective Services Sydney Australia; ^9^ Women's Justice Network Sydney Australia; ^10^ Justice Health and Forensic Mental Health Network Sydney Australia; ^11^ Community Restorative Centre Sydney Australia

**Keywords:** buprenorphine, incarceration, mental disorders, methadone, methamphetamine, opioid dependence, opioids, prison, treatment

## Abstract

**Introduction:**

We have been funded to examine post‐incarceration health and social outcomes for all people incarcerated in New South Wales, Australia, 2000–2022; assess treatment and services for drug dependence and serious mental illness; and project the impact of expanding intervention coverage. We will use a linked cohort, the Prison Outcomes STudy (POST), which we also describe.

**Methods:**

The POST cohort was established using linked administrative data for all adults (≥ 18 years) admitted to full‐time custody in New South Wales, 2000–2022. Custody records were probabilistically linked to 18 health, justice and mortality datasets. We report baseline sociodemographic and custody characteristics and the frequency of key post‐release events.

**Results:**

200,486 adults, 15% women (*n* = 30,698), were incarcerated, with 2,282,367 person‐years of follow‐up and 11% (257,545 person‐years) of follow‐up spent in custody. First Nations people comprised 27% of the cohort. Half (48%) of the cohort (*n* = 95,563) had at least one contact with community mental health services, and 27% (*n* = 54,100) had received alcohol and other drug treatment. POST will provide population‐wide evidence on health and social outcomes after custody, including the effects of treatment for drug dependence and serious mental illness. We will compare across subgroups and the outcomes of post‐release service engagement. Mathematical modelling will test the impact of expanding access to care in prison and post‐release on outcomes in the community.

**Discussion and Conclusions:**

POST will inform policy and service responses across justice, health and community settings to reduce harms among people who experience incarceration.

## 1 Introduction

The global prison population size has increased by 27% since 2000 [1]; over 30 million people are released from prison globally each year [2]. Australian governments spend $6.4 billion each year on prisons ($422 per prisoner per day) [3]. In 2024, there were over 70,000 prison releases in Australia [4], a 36% increase since 2000 [5].

One of the intended goals of incarceration is to punish people for breaking the law, alongside deterrence, rehabilitation and community protection. The population of incarcerated people does, however, include people who are especially susceptible to hardship. This includes First Nations people (in Australia, Aboriginal and Torres Strait Islander people), who comprise 31% of the incarcerated population [6, 7, 8] (compared to 3.2% of the general population [9]); people who use illicit drugs (> 70% of the prison population) [10, 11, 12]; people with serious mental illness (15% of the prison population compared to 1% of the general population) [13, 14, 15, 16]; and women, a growing minority of people in prison (7%) who often come from disadvantaged backgrounds (including domestic violence, childhood trauma and social deprivation).

The negative outcomes associated with incarceration are immense and long‐lasting [17], with increased risks of suicide, fatal drug overdose, communicable and non‐communicable disease, serious mental illness and socio‐economic disadvantage [13, 14, 15, 16]. High reincarceration rates [18] result in re‐exposure to the adverse health and social impacts of prison [19], causing repeated harm to individuals, families and community [20, 21, 22, 23].

Our data linkage study comprises all people incarcerated in New South Wales (NSW) since 2000, linked with 18 state and national administrative datasets. Our National Health and Medical Research Council (NHMRC)‐funded project, which leverages these data, will generate crucial population‐level data on risk of adverse health and social outcomes following release from prison; evaluate the effectiveness of interventions for serious mental illness and drug dependence in reducing adverse outcomes; and model the population‐level impact of increasing coverage of care in prison and linkage to effective interventions post‐release. Despite advances in the management of serious mental illness and drug dependence, significant gaps persist in delivering treatment to people leaving prison. Opioid agonist treatment (OAT; methadone or buprenorphine) for opioid dependence is classified as a World Health Organization essential medicine [24]. Community evidence shows OAT reduces multiple adverse outcomes, including overdose and suicide [25, 26]. We have demonstrated the population‐wide beneficial impacts of OAT among people in NSW [27, 28, 29]. However, there is limited evidence on the uptake and effectiveness of OAT following release from incarceration, including newly introduced long‐acting injectable buprenorphine formulations. Available evidence suggests that maintaining OAT in the community after release is associated with higher rates of primary healthcare contact [30] and lower rates of ambulance contact [31]. Similarly, evidence for the uptake and impact of interventions for methamphetamine dependence post‐release is almost non‐existent [32, 34, 35]. Interventions for serious mental illness, for example, antipsychotic medication, may reduce suicidality, mortality and crime among people with serious mental illness [32, 34, 35], but there is limited research on the extent and impact of linkage to care post‐release among people who have been incarcerated.

This NHMRC‐funded project has three aims:

1. Characterise and quantify adverse health and social outcomes among people released from prison (including mental health, overdose, recidivism and mortality). We will also examine variation in risks among key subpopulations that are rarely studied using other research designs because of the more limited sample sizes that are typically obtained from studies that do not capture the entire population.
2. Assess the extent and impact of interventions targeting opioid and methamphetamine dependence and serious mental illness on adverse health and social outcomes.
3. Use mathematical modelling to assess the potential impact of scaling up mental health and drug dependence interventions on reducing adverse outcomes following release from prison.

## 2 Methods

### 2.1 Ethics Statement

Ethics approvals include NSW Population and Health Services Ethics Committee (No: 2022/ETH00289, including a waiver of consent), Australian Institute of Health and Welfare (No: EO2022/5/1371), Corrections Health (No: 2021.61), Aboriginal Health and Medical Research Council Ethics Committee (No: 1999/22) and UNSW Sydney Human Research Ethics Committee (No: iRECS6272).

### 2.2 Cohort Description

This is a population‐based retrospective cohort study of all adults (≥ 18+ years) with a record of incarceration in NSW between 1 January 2000 and 31 December 2021, recorded in the NSW Bureau of Crime Statistics and Research's Reoffending Database (ROD).

ROD is an internally linked dataset of finalised legal actions within the NSW Criminal Justice System, including all finalised court appearances in the Children's, Local, District and Supreme Courts of NSW since 1994 and all adult episodes of full‐time incarceration to custody since 2000, which have been supplied to the Bureau of Crime Statistics and Research by Corrective Services NSW from their electronic Offender Integrated Management System. The internal matching process between court and custody data within the ROD database has been previously validated and has a specificity of 99.9% and a sensitivity of 93.8% [25]. We have previously used this database in successful data linkage projects [26].

**FIGURE 1:**
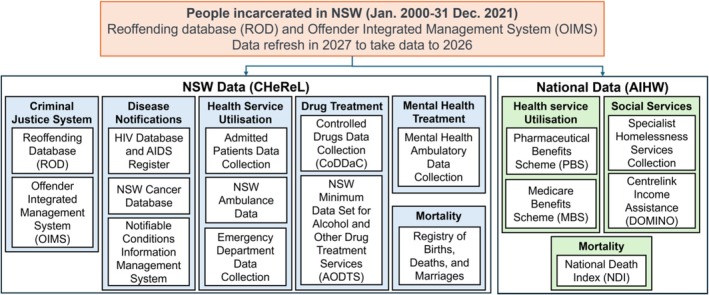
Datasets linked in POST, including date ranges of available data. Abbreviations: AIHW, Australian Institute of Health and Welfare; NSW, New South Wales.

Data for cohort members were linked to 18 state and national datasets to provide content data on health service utilisation, disease notifications, alcohol and other drug treatment, mental health treatment, social services and mortality, in addition to reoffending content data contained in ROD (see Figure 1 and Table 1). Please see Appendix A for details of coding for several key datasets (Tables A1, A2, A3, A4, A5, A6).

**TABLE 1:** Contents of datasets linked to the POST cohort.

Dataset	Contents	Dates available
Reoffending Database (court and custody data)	Court appearances, juvenile detention and adult incarceration in NSW. Contains: criminal offences, court outcomes, mental health dismissals, domestic violence flags, Prescribed Concentration of Alcohol range, drug types, Magistrate's Early Referral Into Treatment (MERIT) program involvement and Level of Service Inventory‐Revised.	Jan 2000–Dec 2021
NSW Offender Integrated Management System	Offender admissions, releases, schedules, movements, classification, property, legal orders, sentence administration and offences in custody	Jan 2000–Dec 2021
NSW Admitted Patients Data Collection	All admitted patient services provided by public hospitals (including psychiatric hospitals), private hospitals and private day procedures centres in NSW. Contains: principal and other diagnoses for hospital stay, episode dates, diagnoses, procedures, health insurance, payment status, demographic and geographic variables.	Jul 2001–Dec 2022
NSW Emergency Department Data Collection	Patient presentations to EDs of public hospitals. Contains: triage category; clinical Information: principal diagnosis, procedures performed, outcome of the visit; hospital Information: hospital identifiers. The number of contributing EDs has increased over time, such that data for more recent years capture a greater proportion of presentations. Previous analyses of ED use in this population have established that data from 2012 onwards provide reasonably complete capture [36].	Jan 2005–Dec 2022
NSW Ambulance Service Data	Records emergency and urgent episodes of care for NSW Ambulance patients who: are transported to a hospital; are left at a scene following clinician assessment; or, who died at the scene. Contains: time and date of call, priority level, incident location, symptoms reported, treatments provided and outcome.	Jan 2009–Dec 2022
NSW Mental Health Ambulatory Data Collection	Records mental healthcare contacts with non‐admitted patients, including mental health day programmes, outreach services, community health service contacts, and outpatient psychiatric contacts. Contains: non‐admitted mental healthcare; service details: dates, type of service, setting, diagnosis codes, treatment modalities used, outcome of the treatment episode; provider Information: type of professional.	Jan 2001–Jun 2022
NSW Minimum Data Set for Alcohol and Other Drug Treatment Services	All specialist services provided to people with alcohol and other drug or gambling problems through government and non‐government drug and alcohol services receiving NSW Ministry of Health funding. Contains: demographic variables, living arrangement, occupation, principal drug, usage method, injecting status, service delivered, delivery setting, service dates, reason for cessation, referrals and comorbidity.	Jan 2015–Dec 2022
NSW Controlled Drugs Data Collection	Data on all people who have received opioid agonist treatment in NSW. Contains: demographic variables, application type, authorised drug, primary opioid, other drugs of concern, maximum dose, prescriber, practice, administration point and reason for cessation.	Jan 1985–Nov 2022
NSW Notifiable Conditions Information Management System	Diagnoses of certain infectious diseases (including hepatitis B and C) and adverse events following immunisation, notified to the NSW Ministry of Health. Contains: condition, date of notification, calculated onset date, demographic variables, geographic variables.	Jan 1993–Mar 2022
NSW HIV Database and the NSW AIDS Register	HIV notifications. Contains: age, health status at diagnosis, date of positive diagnosis and clinical variables describing disease progression.	Jan 1997–Dec 2021
NSW Cancer Registry	Cancer notifications. Contains: type of cancer, date of diagnosis and cancer stage at diagnosis.	Jan 1971–Dec 2020
NSW Registry of Births, Deaths and Marriages	Death registration records date of deaths recorded in NSW.	Jan 2000–Dec 2022
NSW Cause of Death	Cause of Death Unit Record File: records cause of death for deaths recorded in NSW. Contains: cause of death: primary and contributing causes of death; geographical information: residential address at the time of death.	Jan 2000–Dec 2021
National Medicare Benefits Scheme	Claims for all medical and hospital services subsidised by the Commonwealth including doctor visits, pathology tests and imaging	2002—
National Pharmaceutical Benefits Scheme (PBS)	Dispensing records for all PBS‐listed medicines for which the Commonwealth pays a subsidy. Contains: ATC codes, PBS item numbers, drug types; benefit and costing Information: benefit amount; provider and pharmacy details: prescriber speciality, pharmacy identifier (scrambled); Closing the Gap variables (CTG co‐payment scheme).	2002—
National Specialist Homelessness Services Collection	People who present to a Specialist Homelessness Services agency requesting services across Australia. Contains: closed support periods; social and economic status: employment, living arrangements, history of domestic violence; service interaction: types of assistance provided, referral sources, outcomes of service engagements, type of service providers, location of service providers.	2011—
Department of Social Services Centrelink Income Assistance Data (DOMINO)	Event‐based data on individuals' interactions with social security payments and Department of Social Services‐managed programs over time across Australia. Contains: records of interactions with the Department of Social Services and social security payments for individual welfare recipients, benefits history, concessions, education (where available) and housing.	2001—
Medicare Consumer Directory	Medicare enrolment data for all persons enrolled in Medicare.	1984—
National Deaths Index	Deaths recorded in Australia. Contains: cause of death: primary and contributing causes of death.	2000—

*Note:* Dark grey indicates national datasets.

Abbreviations: ED, emergency department; NSW, New South Wales.

### 2.3 Data Linkage and Data Security

Data linkage was conducted by the Centre for Health Record Linkage for NSW datasets and the Australian Institute of Health and Welfare data integration unit for National datasets. Data were linked probabilistically, in accordance with standard models [37]. Personal identifying data used for the purposes of data linkage remain with the data linkage authorities and were not made available to study investigators. Linked content data, identified only by a Person‐Project‐Number, are stored in and accessed via the Secure Unified Research Environment, a high‐security data environment approved by all data custodians and ethics committees. Access to project data is limited to approved and named researchers who are conducting analyses and who have the approval of the principal investigator and all ethics committees. The method of reporting for research outputs will ensure that individual participants cannot be identified. To ensure that results relating to Aboriginal and Torres Strait Islander peoples are reported in a culturally appropriate manner, all study outputs are reviewed by an Aboriginal Reference Group.

### 2.4 Data Cleaning

Prior to any analyses, all received data were cleaned, including logic checks, correction of obvious typographical errors, identification of missing data and data entry errors. Where variables are recorded across datasets, comparison of these will be used to determine ‘correct’ values. For example, Indigenous status is known to be poorly recorded in many health datasets [38]; we have elected to code an individual as Aboriginal and/or Torres Strait Islander if they were identified as such in any of the included datasets.

### 2.5 Cohort Characteristics

**FIGURE 2:**
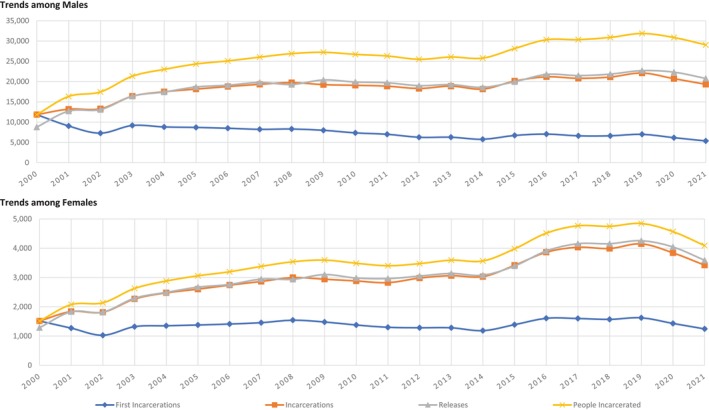
Annual trends in cohort experiencing first incarceration, incarcerated at least once, and released from custody, by sex (2000–2021). *Note:* Total number of men (*n* = 166,238) and women (30,698); Blue = first incarceration during the cohort period (counted once); Orange = individuals incarcerated at least once per year (max once/year); Grey = individuals released from custody per year (max once/year); Yellow = individuals held in custody at any point in the year (counted once/year).

Between 2000 and 2021, 200,486 individuals were incarcerated at least once in NSW (30,698 women and 166,238 men). The number of people who entered the cohort, the number leaving incarceration and the number currently incarcerated each year are presented, by sex, in Figure 2 (see also Table A7 for all data). There was a consistently higher number of men than women experiencing their first adult incarceration during the study period or any incarceration each year, and a similar number of releases from incarceration each year as there were entrances to incarceration. Cohort members spent a total of 257,545 person‐years in incarceration, out of a total of 2,282,367 person‐years of observation (Table 2).

**TABLE 2:** Characteristics of the POST cohort.

	People, *N* (%)	Women, *N* (%)	Men, *N* (%)
Total	**200,486 (100%)**	**30,698 (100%)**	**166,238 (100%)**
Female	**30,698 (15.31%)**		
Male	**166,238 (82.92%)**		
Not reported/other	**3550 (1.77%)**		
Median number of incarcerations (IQR)	2 [1, 4]	2 [1, 3]	2 [1, 4]
Median length of incarceration per custody episode (days; IQR)	5 [1, 104]	1 [1, 41]	7 [1, 121]
% of first incarceration episodes^a^ that included a custodial sentence^b^	40,200 (20.48%)	3842 (12.57%)	36,358 (21.94%)
Median length of incarceration per custody episode that included a custodial sentence (days; IQR)	182 [91–364]	130 [70–252]	182 [96–364]
Median number of charges proven per individual (IQR)	11 [5, 23]	11 [5, 23]	11 [5, 23]
At least one drug offence conviction^c^	82,727 (42.65%)	12,951 (42.98%)	69,775 (42.59%)
At least one property offence conviction^c^	86,434 (44.56%)	16,311 (54.13%)	70,122 (42.80%)
At least one violent offence conviction^c^	137,293 (70.78%)	18,558 (61.58%)	118,734 (72.48%)
Person‐years of observation (after cohort entry)			
Total (years)	2,282,367	320,071	1,919,262
During incarceration (years)	257,545	18,323	237,859
Out of incarceration (years)	2,024,822	301,748	1,681,403
Median age in years [IQR] at cohort entry^c^, ^d^	30 [23, 39]	30 [23, 39]	30 [23, 39]
Year of cohort entry			
2000–2004	53,782 (26.83%)	6505 (21.19%)	46,224 (27.81%)
2005–2009	49,937 (24.91%)	7303 (23.79%)	41,709 (25.09%)
2010–2014	39,811 (19.86%)	6427 (20.94%)	32,722 (19.68%)
2015–2019	42,293 (21.10%)	7793 (25.39%)	34,034 (20.47%)
2020–2021	14,663 (7.31%)	2670 (8.70%)	11,549 (6.95%)
Aboriginal and/or Torres Strait Islander identification^c^	51,648 (25.76%)	11,055 (36.01%)	40,583 (24.41%)
Geographic residence at first incarceration			
Remoteness^e^			
Major cities	129,531 (64.61%)	20,025 (65.23%)	109,504 (65.87%)
Inner regional	37,362 (18.64%)	6281 (20.46%)	31,081 (18.70%)
Outer regional	12,451 (6.21%)	1977 (6.44%)	10,473 (6.30%)
Remote	1705 (0.85%)	304 (0.99%)	1401 (0.84%)
Very remote	539 (0.27%)	120 (0.39%)	419 (0.25%)
Interstate or overseas	4514 (2.25%)	612 (1.99%)	3902 (2.35%)
Incarceration at time of contact	3258 (1.63%)	320 (1.04%)	2938 (1.77%)
Unknown	11,126 (5.55%)	1059 (3.45%)	6520 (3.92%)
Socio‐economic disadvantage^f^:			
1. Most disadvantaged (deciles 1 and 2)	50,449 (25.16%)	7851 (25.57%)	42,597 (25.62%)
2.	38,246 (19.08%)	6124 (19.95%)	32,122 (19.32%)
3.	48,714 (24.30%)	8033 (26.17%)	40,681 (24.47%)
4.	31,939 (15.93%)	4795 (15.62%)	27,142 (16.33%)
5. Least disadvantaged (deciles 9 and 10)	16,604 (8.28%)	2378 (7.75%)	14,226 (8.56%)
Unknown	14,534 (7.25%)	1517 (4.94%)	9470 (5.70%)
% who ever received an HIV notification	685 (0.34%)	64 (0.21%)	617 (0.37%)
% who ever received an HCV notification	26,226 (13.08%)	5734 (18.68%)	20,470 (12.31%)
% who ever received a sexually transmitted infection(s) notification^g^	20,375 (10.16%)	5756 (18.75%)	14,601 (8.78%)
Died during observation period (BDM)	13,394 (6.68%)	1876 (6.11%)	11,518 (6.93%)

*Note:* Due to missing data on sex, values in ‘People’ column will exceed the sum of the Men and Women columns.

Abbreviations: BDM, Births, Deaths and Marriages; HCV, hepatitis C virus; IQR, interquartile range.

^a^ First adult incarceration from 2000 to 2022. Some individuals could have been first incarcerated before 2000.

^b^ Some incarcerations involved remand only.

^c^ Charge data were coded according to the Australian and New Zealand Standard Offence Classification system (see Appendix A); participants with missing charge data (*n* = 6522) were excluded from the denominator in column percentage calculations unless the variable was recorded as not missing within another dataset (see Table 4 for details).

^d^ Age data missing for cohort entry, *n* = 485 (2.42%).

^e^ Using the Accessibility/Remoteness Index of Australia to postcode at first incarceration or 1 year prior to any dataset if missing.

^f^ Using the Socio‐Economic Indexes for Areas to postcode at first incarceration by deciles or 1 year prior to any dataset if missing.

^g^ Chlamydia, gonorrhoea, syphilis and lymphogranuloma venereum.

^e^ Note that people still incarcerated at the end of the follow‐up period had their follow‐up censored at that time point.

Tables 2 and 3 present descriptive characteristics of the cohort. At cohort entry, the median age was 30 years; 26% were identified as being Aboriginal and/or Torres Strait Islander (cf. comprising around 3.2% of the general Australian population). Most people were living in major cities at the time of incarceration (65%); 25% came from the two most economically disadvantaged areas (which contain 20% of the general population).

**TABLE 3:** POST cohort drug and alcohol treatment and other health service contacts.

	People, *N* (%)	Women, *N* (%)	Men, *N* (%)
Any opioid agonist treatment (2001–)	30,091 (15.01%)	6344 (20.66%)	23,745 (14.28%)
During incarceration	20,843 (10.40%)	4302 (14.01%)	16,541 (9.95%)
In the community	29,457 (14.69%)	6298 (21.52%)	23,157 (13.93%)
Drug and alcohol treatment (primary drug) (2015–)^a^			
Alcohol (incl. hospital withdrawal)	14,724 (7.55%)	2719 (9.07%)	12,005 (7.43%)
Opioids	16,084 (8.24%)	3575 (11.92%)	12,509 (7.74%)
Cannabis	11,131 (5.70%)	2416 (8.06%)	8715 (5.39%)
Amphetamines	20,900 (10.71%)	5100 (17.01%)	15,800 (9.78%)
Cocaine	1136 (0.58%)	100 (0.33%)	1036 (0.64%)
Benzodiazepines	1462 (0.75%)	414 (1.38%)	1048 (0.65%)
Psychostimulants	582 (0.30%)	162 (0.54%)	420 (0.26%)
Other	1648 (0.84%)	522 (1.74%)	1126 (0.70%)
Multiple drugs (> 1 of the above)	14,293 (7.32%)	3508 (11.70%)	10,785 (6.67%)
Any mental health contact (ambulatory) (2001–)	95,653 (47.71%)	18,706 (60.94%)	76.843 (46.22%)
Any emergency department presentation (2005–)^b^	162,311 (81.22%)	26,116 (85.29%)	136,192 (82.21%)
Any ambulance callout (2009–)^c^	119,068 (59.96%)	21,808 (71.58%)	97,233 (59.08%)
Ambulance callout for suspected drug overdose (2009–)^c^	28,899 (14.55%)	7216 (23.68%)	21,681 (13.17%)
Hospital stay for overdose/poisoning due to^d^ (ever)			
Alcohol	5050 (2.52%)	1462 (4.76%)	3588 (2.16%)
Opioids	7165 (3.57%)	2033 (6.62%)	5132 (3.09%)
Cannabis	1042 (0.52%)	256 (0.83%)	786 (0.47%)
Amphetamines	3142 (1.57%)	805 (2.62%)	2337 (1.41%)
Cocaine	571 (0.28%)	90 (0.29%)	481 (0.29%)
Multiple drugs (> 1 of the above)	3489 (1.74%)	989 (3.22%)	2500 (1.50%)
Hospital stay for substance use disorders due to^d^ (2001–)			
Alcohol	43,878 (21.89%)	7776 (25.33%)	36,102 (21.72%)
Opioids	21,169 (10.56%)	5704 (18.58%)	15,465 (9.30%)
Cannabis	32,541 (16.23%)	7159 (23.32%)	25,382 (15.27%)
Sedatives	9467 (4.72%)	2770 (9.02%)	6697 (4.03%)
Cocaine	4354 (2.17%)	916 (2.98%)	3438 (2.07%)
Stimulants	27,686 (13.81%)	6653 (21.67%)	21,032 (12.65%)
Hallucinogens	1197 (0.60%)	219 (0.71%)	978 (0.59%)
Hospital stay for infections potentially related to injecting drug use^d^ (2001–)			
Skin or soft tissue infection	27,217 (13.58%)	5493 (17.89%)	21,724 (13.07%)
Endocarditis	1903 (0.95%)	505 (1.65%)	1398 (0.84%)
Osteomyelitis	2557 (1.28%)	451 (1.47%)	2106 (1.27%)
Bacteraemia or sepsis	7428 (3.70%)	1740 (5.67%)	5688 (3.42%)
Central nervous system infections	442 (0.22%)	128 (0.42%)	314 (0.19%)
Septic arthritis	1625 (0.81%)	296 (0.96%)	1329 (0.80%)
Hospital stay for liver‐related causes^d^ (2001–)			
Viral hepatitis	18,166 (9.06%)	4806 (15.66%)	12,914 (7.77%)
Liver cancer	498 (0.25%)	32 (0.10%)	452 (0.27%)
Fibrosis and cirrhosis	1774 (0.88%)	306 (1.00%)	1383 (0.83%)
Hospital stay for HIV‐related causes^d^ (2001–)	482 (0.24%)	49 (0.16%)	418 (0.25%)
Any hospitalisation for^d^			
Psychosis	18,864 (9.41%)	3728 (12.14%)	15,135 (9.10%)
Self‐harm	19,083 (9.52%)	5166 (16.83%)	13,917 (8.37%)
Depression	22,627 (11.29%)	5941 (19.35%)	16,686 (10.04%)
Other mental disorders (excl. substance use disorders)	40,580 (20.24%)	9734 (31.71%)	30,845 (18.55%)
Accidental injuries (excl. overdose)	74,239 (36.33%)	10,438 (34.00%)	63,801 (38.38%)
Assault	27,959 (13.95%)	4854 (15.81%)	23,105 (13.90%)

^a^ As recorded in the NSW Minimum Data Set for Alcohol and Other Drug Treatment Services, see Table A3 (cohort denominator from 2015: total = 195,132, *F* = 29,982, *M* = 161,600).

^b^ Cohort denominator from 2005: total = 199,830, *F* = 30,621, *M* = 165,659.

^c^ Based on ambulance callouts for suspected drug overdose, identified through specific callout types, see Table A4 (cohort denominator from 2009: total = 198,582, *F* = 30,467, *M* = 164,565).

^d^ Hospital diagnoses are based on the appearance of the specific diagnosis codes appearing in the principal or contributing diagnosis fields. ICD‐10 codes for each category are provided in Table A5.

Approximately half of the cohort (53.5%) were incarcerated for periods on remand only (i.e., were not incarcerated following a custodial sentence); 26.7% had one episode of incarceration following sentencing, and 19.9% had more than one. Men in the cohort had higher proportions of sentenced episodes: 27.7% had one, and 21.0% had more than one, compared with 20.9% and 13.5% among women in the cohort. The median number of incarcerations during follow‐up was 2 (interquartile range [IQR]: 1, 4); most incarcerations (including remand) were for short durations (median 4 days [IQR: 1, 104 days]). The median number of proven criminal charges during the observation period was 11 (IQR: 5, 24); 71% of the cohort had at least one violent offence conviction, and 4 in 10 had at least one drug offence or property offence.

Levels of HIV notifications were very low in the cohort (0.34%); just over one in eight people in the cohort received a notification of hepatitis C infection at some point (13.8%). One in 10 received a notification of a sexually transmitted infection at some point (10.6%). Using data from the NSW Registry of Births, Deaths and Marriages, 6.8% of the cohort died during follow‐up (13,394 people; Table 2).

Table 3 presents information on some health service use of the cohort (recorded at any time in each of the datasets). Opioid agonist treatment (methadone or buprenorphine) was accessed at some point by 15.01% of the cohort (with slightly higher levels among women). One in 10 had ever accessed community drug treatment for amphetamine use (10.7%; 17.0% among women, 9.9% among men).

The majority (81%) had at least one emergency department visit, and most (60%) had an ambulance callout. Ambulance callouts for suspected overdoses had occurred for 23.2% of women and 12.82% of men. Outpatient mental health treatment had been accessed by almost half of the cohort (47.7%).

Hospital stays for substance use disorders were coded as one of the reasons for hospital stays for a substantial minority (e.g., 22.0% for alcohol use disorders; 13.8% for stimulant use disorders and 10.6% for opioid use disorders). Almost 1 in 10 had experienced a hospital stay for psychosis (9.4%) or self‐harm (9.5%). Almost 1% (0.95%) had ever had a hospital stay related to endocarditis, and 13.6% for a skin or soft tissue infection.

## 3 Results

### 3.1 Aim 1: Quantify Adverse Health and Social Events Post‐Release

We will quantify rates of adverse health and social events (Table 3) among people released from incarceration for the study follow‐up time (or until reincarceration or death) and at different time periods (1‐, 3‐, 6‐, 12‐, and 24‐month post‐release), with confidence intervals derived from a Poisson or negative binomial distribution as appropriate. We will test for differences in outcomes among specific subgroups (Table 4), including individuals with histories of serious mental illness, opioid dependence, methamphetamine dependence, women and First Nations people.

**TABLE 4:** Health and social outcomes, and subgroup operationalisation.

Outcomes	Examples of sources and definitions that may be operationalised
Non‐fatal opioid, other drug overdose	*APDC* (Table 1): ICD‐10 poisoning *EDDC* (codes reported in [20])
Self‐harm hospitalisations	*APDC*: ICD‐10 self‐injury (X60–X69; X70–X79; X80–X84; Y10–Y34 and Y87.0) [22]
Injection‐related injuries and diseases	*APDC, EDDC*: including abscess, cellulitis, endocarditis and other infections (codes in [33])
Acute mental health problems	*APDC*: ICD‐10; *EDDC; Mental Health Ambulatory Data Collection*
Fatal accidental overdose	*National Deaths Index (NDI)*: ICD‐10 poisoning (codes reported in [20])
Suicide	*NDI*: ICD‐10 self‐injury (X60–X69; X70–X79; X80–X84; Y10–Y34 and Y87.0) [22]
All‐cause mortality	*NDI*: all deaths
Reoffending	*ROD*: Property, drug‐related and violence charges (codes reported in [35, 55])
Reincarceration	*ROD*: Custody records
Homelessness	*SHSC*: Homelessness services; *APDC*: ICD‐10 diagnoses code
Unemployment	*DOMINO*: Centrelink Income Assistance

Subgroups	Examples of source and definitions that may be operationalised
Women	Identified through sex variables in any of the linked datasets
First Nations people	Identified through Indigenous status in any of the linked datasets

Abbreviations: APDC, Admitted Patients Data Collection; DOMINO, Department of Social Services Centrelink Income Assistance Data; EDDC, Emergency Department Data Collection; ROD, Reoffending Database; SHSC, Specialist Homelessness Services Collection.

Using extended survival and generalised linear regression models, we will calculate effect estimates (e.g., hazard ratios, odds ratios and incidence rate ratios) to assess heterogeneity between population subgroups descriptively, by constructing models within each subgroup, and formally through hypothesis testing of interaction terms. Analyses will be stratified by duration of incarceration, consider each adverse outcome separately and be adjusted for relevant confounders (Table 4 shows examples). We will also investigate the effect of repeat incarceration episodes by using methods that incorporate multiple observations per person (e.g., generalised linear mixed models and generalised estimating equations). The generalised estimating equation approach will account for the correlated nature of repeated measurements among individuals; logistic models will be used to calculate the odds of an outcome; Poisson models will calculate the rates (the negative binomial distribution will be considered in the presence of overdispersion). Cox regression models will be utilised to calculate the median time to health and social outcomes from release from incarceration. Measures of association will be estimated using a competing risk model (e.g., Fine–Grey model) when competing risks are present, such as when death occurs that is not the outcome of interest, for example, when estimating the time to suicide and death occurred due to other circumstances. Adjustments will be made using the same confounders as for the generalised estimating equation approach. Weighted Schoenfeld residuals will be used to examine the assumption of proportional hazards. Subgroup analyses will focus on individuals with histories of opioid/methamphetamine dependence or serious mental illness; we will examine potential differences in outcomes for women and First Nations people and consider other subpopulations such as those who have experienced homelessness. These data critically inform Aims 2 and 3.

Far smaller studies than ours, with much shorter follow‐up [39], have had sufficient power to show associations between sex and mental disorders when examining overdose and mortality (rarest outcomes). We will have sufficient power for all subpopulations: to detect a 25% difference in mortality between males and females (the smallest subgroup), 12 months post‐release would require ≈ 17,923 females and ≈ 118,292 males (female mortality rate of 0.008; male rate of 0.0102; 80% power; 5% significance; 6.6 ratio of males/females) [15]. Power to examine differences among First Nations people will be higher [40].

### 3.2 Aim 2: Assess the Effect of Interventions to Reduce Adverse Health and Social Events Among Select Subpopulations

Aim 2 analyses will assess the impact of interventions on the risk of adverse health and social outcomes. We will examine interventions provided to people released from prison with a history of opioid dependence, methamphetamine dependence or serious mental illness. We will test for variation in exposure and treatment effects between women and men, and between Aboriginal and Torres Strait Islander peoples and non‐Indigenous people. Interventions for opioid dependence include methadone, buprenorphine and long‐acting injectable buprenorphine; interventions for methamphetamine dependence will include counselling, support, case management and residential rehabilitation. Interventions for serious mental illness will include visits with a psychologist, psychiatrist or mental health outpatient, and relevant medications such as antipsychotics. Periods in and out of treatment will be estimated.

The overall objective of the NHMRC‐funded study is to assess the impact of interventions on the risk of adverse health and social outcomes immediately upon leaving incarceration. We will examine interventions provided to three subpopulations—people with opioid dependence, methamphetamine dependence and SMI (Table 5). For people with a history of each disorder, we will investigate the time taken to obtain disorder‐specific treatment in the community, censoring at the end of follow‐up, death or reincarceration. We will test for variations in exposure and treatment effects between women and men, and between First Nations and non‐First Nations people, using similar approaches described in Aim 1.

**TABLE 5:** Operationalisation of services being considered, and potential covariate operationalisation.

Interventions	Source and potential operationalisation
Opioid dependence treatment (exposure as defined in [20, 22, 28, 33, 34])	Several formulations of opioid agonist and partial opioid agonist treatment (OAT) have been prescribed in New South Wales: methadone; buprenorphine (daily dosage); and from 2018, long‐acting injectable buprenorphine (weekly and monthly dosage). The medical authority applications to prescribe OAT are recorded in the CoDDaC and will be used to determine periods of OAT both within the incarceration period and continuation in the community.
Methamphetamine dependence treatment (as defined in [42])	Treatments for methamphetamine dependence are recorded in the AODTS. We will examine residential rehabilitation, counselling, support and case management.
Treatment for serious mental illness (see [43])	Mental health outpatient visit records (MH‐AMB) and MBS mental health service records for care for SMI in the community (e.g., MBS items from A8 Consultant psychiatrist attendances A15 Multidisciplinary care plans); PBS medication dispensings (e.g., antipsychotics: clozapine, risperidone, olanzapine and quetiapine).

Example covariates	Source and examples of definitions to be operationalised
Sociodemographic	Age, geographic remoteness, sociodemographic and economic disadvantage status
Extent of comorbidity	*APDC*: hospitalisation for chronic diseases (liver, kidney, respiratory, circulatory), CANCER, *NCIMS*: HIV
Mental health history	*MH‐AMB, MBS, PBS, EDDC, APDC*: Treatment for mental disorders with medicines, mental healthcare plans, outpatient care or hospital admission
History of suicidality	*EDDC, APDC*: prior visit for attempted suicide.
History of substance dependence	*MH‐AMB*: substance dependence treatment; *EDDC/APDC*: prior alcohol or illicit drug‐related admissions; AODTS: treatment for alcohol and other drugs
Criminogenic variables	*ROD*: classification of prior criminal charge(s) (property, drug and\or violent), previous incarceration; length of incarceration
Welfare payments	*DOMINO*: Department of Social Services Centrelink income assistance
History of homelessness	*SHSC*: Homelessness services

*Note:* For all acronyms and details of datasets, see Table 1.

Abbreviations: AODTS, Alcohol and Other Drug Treatment Services; APDC, Admitted Patients Data Collection; CoDDaC, Controlled Drugs Data Collection; EDDC, Emergency Department Data Collection; LAIB, long‐acting injectable buprenorphine; MBS, Medicare Benefits Scheme; MH‐AMB, Mental Health Ambulatory Data Collection; NCIMS, Notifiable Conditions Information Management System; PBS, Pharmaceutical Benefits Scheme; ROD, Reoffending Database; SHSC, Specialist Homelessness Services Collection.

### 3.3 Aim 3: Modelling the Population‐Level Impact of Scaling Up Interventions Post‐Release

We will use mathematical modelling to project the population‐level impacts of scaling up mental health, opioid and methamphetamine dependence interventions post‐release. We will assess the impact achieved by past intervention provision and estimate the potential reductions in adverse outcomes (see Table 4) that could be achieved by scaling up linkage to care upon release. Impact will be measured, overall and by sex and First Nations status, and at the intersection of sex and First Nations status, in terms of the number of adverse events averted (e.g., drug‐related deaths and suicide) and the relative reduction in incidence of adverse outcomes.

Leveraging our experience with dynamic modelling of OAT's impact on mortality [25, 44], we will develop an individual‐based model of the cohort subpopulations with opioid dependence to estimate the population‐level impact of post‐release OAT. The models will simulate the trajectory of health, treatment, and social outcomes of each individual within the cohort. Using cohort data, individuals will be assigned characteristics such as age, sex, First Nations status and other key covariates identified in Aim 1. Upon release, individuals will receive each intervention with probabilities informed by cohort data, which can differ based on their characteristics and over time.

Informed by Aims 1 and 2, individuals will experience adverse health and social outcomes with probabilities that are dependent on their characteristics, intervention exposure and time since prison release, including any interactions (e.g., differential exposure and/or effectiveness of interventions by First Nations status or sex). The model will be used to estimate the impact of interventions over 2000–2021 by comparing baseline model simulations with counterfactual scenarios in which interventions are not delivered, similar to our previous modelling for all people with opioid dependence [25, 44]. Impact will be measured in terms of adverse events averted (e.g., drug‐related deaths and suicide) and the relative reduction in incidence of adverse outcomes. Analyses will also estimate the differential impact of these interventions for First Nations people and women, resulting from differential baseline risks of adverse events and uptake and/or effectiveness of OAT.

Analyses of cohort data will identify trends in the number of people with opioid dependence who are incarcerated for the first time and their characteristics. Projecting forward, the model will then be used to estimate the impact of different scenarios which scale up specific post‐release interventions, informed by our advisory groups. Analyses will identify which intervention scenarios maximise impact for the overall population, and among First Nations people and women.

In a similar way, we will develop individual‐based models of the cohort subpopulations with methamphetamine dependence and SMI to evaluate the impact of post‐release interventions and of scaling‐up interventions targeted to these subpopulations going forward, if we generate evidence of effectiveness for specific interventions. If interventions for SMI and methamphetamine dependence show no/limited effectiveness in Aim 2, we will conduct scenario analyses that model interventions with different effectiveness on outcomes based on consultations with the Community Advisory Group, First Nations Reference Group (FNRG), other external stakeholders and international evidence [45]. Combining sub‐models for opioids, methamphetamine and SMI, we will then develop an overall model of the entire cohort population updated to 2000–2024 data and incorporating results from Aim 2.4. We will compare the incidence rate ratio of adverse outcomes by sex and First Nations status with and without the scale‐up.

### 3.4 Relevance for Consumers and Community

We address many government priorities. This project is aligned with the National Mental Health and Suicide Prevention Plan (*Priority 3: Coordinating treatment and supports for people with severe and complex mental illness; Priority 4—improving First Nations mental health and suicide prevention*) and the Australian Government's *National Drug Strategy 2017–2026*, where priority populations are people in contact with the criminal justice system and First Nations people. It also aligns with the *National Agreement on Closing the Gap Plan* [45], with targets on reducing incarceration and suicide among First Nations people.

The project will be informed by the insights of individuals with lived experience of incarceration through the FNRG and the Community Advisory Group, as well as community partners. Guidance from advocacy groups for people who experience incarceration, the FNRG for our project, and other key stakeholders have shaped the core research questions and informed the project's analyses for women and First Nations people. We will continue to co‐design the project with advisory groups using a structured framework to continuously improve our research questions, critique methodologies, interpret findings and guide effective dissemination.

### 3.5 Data Resource Access

To protect privacy and confidentiality, approval for the linkage of health data in NSW is provided under strict conditions for the storage, retention and use of the data. The current approval permits storage of the data at one site, the University of New South Wales, Sydney, for up to 7 years following the date of publication of results. Data may only be supplied for analysis within Australia.

We encourage interested parties to contact us to discuss potential secondary data analyses, noting that legislation requires that the data can only be stored and analysed within NSW. Virtual Private Network and other virtual access are not permitted under current approvals. Requests for data access can be submitted to Professor Louisa Degenhardt (l.degenhardt@unsw.edu.au) and will be reviewed by the POST investigator team. Potential collaborators will be required to gain approval for data access and specific secondary analyses from the NSW Population and Health Services Research Ethics Committee. Collaborators proposing to examine research questions relating specifically to Aboriginal peoples will also be required to seek approval from the Aboriginal Health and Medical Research Council.

## 4 Discussion

Although studies have documented the elevated risk of communicable diseases and mortality in prisoners post‐release [46], most health and social outcomes remain under‐researched [47], and there is a lack of population‐wide research on health and social outcomes post‐release from incarceration. Individuals with serious mental illness or drug dependence, women and First Nations people face unique challenges and health disparities that are not adequately addressed by general population studies [7, 48, 49, 50]. Despite this, research on these subpopulations among people who are incarcerated is limited: evidence to drive change and improve outcomes is critically needed.

Policymakers and those working with people who are incarcerated are acutely aware of the risks faced by people released from incarceration, but strong population evidence on interventions to reduce these risks is lacking. We have shown that the impacts of mental health and drug dependence treatment after release from incarceration are neither well studied nor understood [27]. For example, only two studies have examined the impact of opioid agonist treatment (OAT, methadone or buprenorphine, classified as World Health Organization essential medicines [24]) delivered during incarceration on mortality post‐release [28], and only one of those examined linkage to OAT provided post‐release [28, 29]. A long‐acting injectable buprenorphine formulation (delivered weekly/monthly) for the treatment of opioid dependence, found to be effective in the community [51, 52], has recently been introduced and rapidly scaled up in prisons in Australia [53], but there has been no evaluation of potential differences in outcomes for people released from incarceration on this new buprenorphine formulation compared to methadone. This is important because there is evidence to suggest that people may prefer methadone to buprenorphine (but are given limited choice of OAT medication during incarceration, and are typically required to receive long‐acting injecting buprenorphine [53]), and therefore may be more likely to cease buprenorphine after leaving prison. This would potentially increase risks of the adverse outcomes that are known to be highly elevated for opioid dependent people post‐release, particularly mortality [54].

There has been no evaluation of the impact of either methamphetamine dependence or serious mental illness treatment provided to people released from incarceration in Australia. Additionally, no study has examined potential subpopulation differences of post‐release interventions for mental health and drug dependence [25]. And importantly, no studies have considered comorbidity between drug dependence and serious mental illness in terms of impact on outcomes post‐release and the impact of interventions to dually treat these disorders.

Our study will fill these gaps using real‐world data. Our study will use a population‐wide linked cohort to quantify the risk of post‐release harms, including Amon key subpopulation, and assess the effectiveness of interventions following release from prison. It will be the first to jointly examine how serious mental illness and drug dependence contribute to these outcomes. Findings will underpin mathematical modelling, which will identify where improved linkage to care post‐release can yield the greatest benefits. These findings will inform policy and practice across justice, health, and community sectors. This work will quantify outcomes and generate evidence around critical points for intervention to reduce health and social harms among people released from incarceration, informing Justice Health, Corrective Services and community agencies. It will also answer policy‐relevant questions about the impact of improving the transition from incarceration to community care; and additionally, the benefits of increasing programmes that divert people from incarceration.

### 4.1 Strengths and Weaknesses

A key strength of this study is the use of the NSW ROD as a sampling frame, capturing all people in NSW who have been incarcerated on any given day since 2000, eliminating coverage error. As individuals in the cohort have data linked from multiple longitudinal health and social data collections, POST serves as a growing resource for a range of study designs and research questions relating to incarceration, as well as to related interventions and policies.

As these data collections are primarily collected for administrative purposes, the data are limited to those variables that are routinely collected. We have notification data on HIV and HCV infection, for example, but this will underestimate true prevalence if not all people who have been incarcerated have been tested. Data on indicators of so cial determinants of health are available, such as homelessness and socio‐economic status—the latter of which is inferred indirectly by applying the Socio‐Economic Indexes for Areas to the postcode listed for each participant [37]. Another limitation concerns our limited capacity to examine health services delivered during incarceration: many services, such as prescribed medicines and general medical services, are not contained within the routine datasets collected for community‐delivered healthcare (PBS and Medicare Benefits Scheme, specifically), limiting our capacity to assess exposure to some health interventions during incarceration (Tables A1, A2, A3, A4, A5, A6, A7).

Despite these limitations, the size and duration of the cohort will permit analyses of rare adverse outcomes and changes over time. We have linked multiple health and criminal justice datasets that provide a rich set of covariates for inclusion in predictive models. These data will enable detailed exploration of trajectories that account for individual and treatment setting variables and how these may influence outcomes. We expect findings from these analyses to inform clinical guidelines for care during incarceration and post‐release.

## Author Contributions

L.D. and T.S. led the writing of the paper. T.S. led and supervised analyses of the POST cohort study that feature in this manuscript. All authors made substantial contributions to the critical review, editing and revision of the manuscript. All authors approved the final version of the manuscript.

## Funding

This work was supported by the Australian NHMRC through a program grant (the ASCEND program: Advancing the health of people who use drugs: hepatitis C and drug dependence [ASCEND], the National Drug and Alcohol Research Centre), an NHMRC Clinical Trials and Cohort study grant (#2041832; the Prison Outcomes Study [POST]) and leveraging an NIH project grant (R01 DA144740, R01 DA059822). L.D. is supported by an NHMRC Investigator Award Level 3 (#2016825). The National Drug and Alcohol Research Centre is supported by funding from the Australian Government Department of Health under the Drug and Alcohol Program. M.H. and P.V. acknowledge support from NIHR HPRU in Behavioural Science and Evaluation and NIHR Programme Grant EPIToPe. P.V. and J.S. acknowledge support from the Wellcome Trust (WT 220866/Z/20/Z and 226619/Z/22/Z).

## Conflicts of Interest

In the past 3 years, G.D. has received research grants from Gilead, AbbVie and Merck. The authors declare no conflicts of interest.

## Data Availability

The data are not publicly available due to privacy or ethical restrictions.

## Appendix A

**TABLE A1:** Variables derived from multiple datasets.

Condition	Source datasets
Sex	ROD, CoDDaC, EDDC, APDC, MH, NCIMS, MDS
Postcode 1 year prior to incarceration	ROD, CoDDaC, EDDC, APDC, MH, NCIMS, MDS
Indigenous status	ROD, CoDDaC, EDDC, APDC, MH, NCIMS, MDS

Abbreviations: APDC, Admitted Patients Data Collection; CoDDaC, Controlled Drugs Data Collection; EDDC, Emergency Department Data Collection; MDS, Minimum Data Set; MH, Mental Health; NCIMS, Notifiable Conditions Information Management System; ROD, Reoffending Database.

**TABLE A2:** Approach to coding of criminal charges. Charge data were coded according to the Australian and New Zealand Standard Offence Classification (ANZSOC) system. This has 16 major categories of offence. Our definition of *violent offences* was consistent with that used by the Bureau of Crime Statistics and Research (BOCSAR) in its standard crime statistic reporting [41] and included: Murder (ANZSOC codes 011, 012, 013); assault (021, 029), robbery (0611, 0612), sexual assault and indecent assault/act of indecency/other sexual offences (0311, 0312). Similarly, we used the standard BOCSAR definition of *property offences,* which included: Break and enter (071), motor vehicle theft (081), theft (082) and fraud (091, 092).

01 Homicide and related offences.
02 Acts intended to cause injury.
03 Sexual assault and related offences.
04 Dangerous or negligent acts endangering persons.
05 Abduction, harassment and other offences against the person.
06 Robbery, extortion and related offences.
07 Unlawful entry with intent/burglary, break and enter.
08 Theft and related offences.
09 Fraud, deception and related offences.
10 Illicit drug offences.
11 Prohibited and regulated weapons and explosives offences.
12 Property damage and environmental pollution.
13 Public order offences.
14 Traffic and vehicle regulatory offences.
15 Offences against justice procedures, government security and government operations.
16 Miscellaneous offences.

*Note:* Pink B. Australian and New Zealand Standard Offence Classification (ANZSOC), 3rd ed. http://www.ausstats.abs.gov.au/Ausstats/subscriber.nsf/0/5CE97E870F7A29EDCA2578A200143125/$File/12340_2011.pdf.

**TABLE A3:** Condition codes for classification of sexually transmitted infections.

Condition	Condition code
Chlamydia	CHLAM
Chlamydia—Congenital	CHLAMCONG
Gonorrhoea	GONOR
LGV	LGV
Syphilis—Infectious	SYPH
Syphilis—Congenital	SYPHCONG
Syphilis—> 2 years or unknown duration	SYPHUN

**TABLE A4:** Australian standard classification of drugs of concern categories.

Principal drug/gambling description	Category	Code
Inadequately described	Inadequately described	0000
Not stated	Inadequately described	0001
Gambling	Other drugs of concern	0002
None/no other drugs of concern	Inadequately described	0003
Pharmaceutical opioids, nfd	Opioid analgesics (new category)	0005
Psychostimulants, nfd	Psychostimulants, nfd	0006
Gambling	Other drugs of concern	0009
Analgesics, nfd	Opioid analgesics (new category)	1000
Organic opiate analgesics, nfd	Opioid analgesics (new category)	1100
Codeine	Opioid analgesics (new category)	1101
Morphine	Opioid analgesics (new category)	1102
Organic opiate analgesics, nec	Opioid analgesics (new category)	1199
Semisynthetic opioid analgesics, nfd	Opioid analgesics (new category)	1200
Buprenorphine	Opioid analgesics (new category)	1201
Heroin	Opioid analgesics (new category)	1202
Oxycodone	Opioid analgesics (new category)	1203
Buprenorphine/naloxone	Opioid analgesics (new category)	1298
Semisynthetic opioid analgesics, nec	Opioid analgesics (new category)	1299
Synthetic opioid analgesics, nfd	Opioid analgesics (new category)	1300
Fentanyl	Opioid analgesics (new category)	1301
Fentanyl analogues	Opioid analgesics (new category)	1302
Levomethadyl acetate hydrochloride	Other drugs of concern	1303
Meperidine analogues	Other drugs of concern	1304
Methadone	Opioid analgesics (new category)	1305
Pethidine	Opioid analgesics (new category)	1306
Tramadol	Opioid analgesics (new category)	1307
Synthetic opioid analgesics, nec	Opioid analgesics (new category)	1399
Non‐opioid analgesics, nfd	Other drugs of concern	1400
Paracetamol	Other drugs of concern	1402
Ibuprofen	Other drugs of concern	1403
Non‐opioid analgesics, nec	Other drugs of concern	1499
Sedatives and hypnotics, nfd	Other drugs of concern	2000
Alcohols, nfd	Alcohols, nfd	2100
Ethanol	Alcohols, nfd	2101
Methanol	Alcohols, nfd	2102
Alcohols, nec	Alcohols, nfd	2199
Anaesthetics, nfd	Other drugs of concern	2200
Gamma‐hydroxybutyrate (pre‐2015), replaced by 2501	Other drugs of concern	2201
Ketamine	Other drugs of concern	2202
Nitrous oxide	Other drugs of concern	2203
Phencyclidine	Other drugs of concern	2204
Propofol	Other drugs of concern	2205
Anaesthetics, nec	Other drugs of concern	2299
Barbiturates, nfd	Other drugs of concern	2300
Amylobarbitone	Other drugs of concern	2301
Methylphenobarbitone	Other drugs of concern	2302
Phenobarbitone	Other drugs of concern	2303
Barbiturates, nec	Other drugs of concern	2399
Benzodiazepines, nfd	Benzodiazepines, nfd	2400
Alprazolam	Benzodiazepines, nfd	2401
Clonazepam	Benzodiazepines, nfd	2402
Diazepam	Benzodiazepines, nfd	2403
Flunitrazepam	Benzodiazepines, nfd	2404
Lorazepam	Benzodiazepines, nfd	2405
Nitrazepam	Benzodiazepines, nfd	2406
Oxazepam	Benzodiazepines, nfd	2407
Temazepam	Benzodiazepines, nfd	2408
Benzodiazepines, nec	Benzodiazepines, nfd	2499
GHB‐type drugs and analogues, nfd	Other drugs of concern	2500
Gamma‐hydroxybutyrate	Other drugs of concern	2501
Gamma‐butyrolactone	Other drugs of concern	2502
1,4‐butanediol	Other drugs of concern	2503
GHB type drugs and analogues, nec	Other drugs of concern	2599
Other sedatives and hypnotics, nfd	Other drugs of concern	2900
Kava lactones	Other drugs of concern	2902
Zopiclone	Other drugs of concern	2903
Doxylamine	Other drugs of concern	2904
Promethazine	Other drugs of concern	2905
Zolpidem	Other drugs of concern	2906
Other sedatives and hypnotics, nec	Other drugs of concern	2999
Stimulants and hallucinogens, nfd	Amphetamines, nfd	3000
Amphetamines, nfd	Amphetamines, nfd	3100
Amphetamine	Amphetamines, nfd	3101
Dexamphetamine	Amphetamines, nfd	3102
Methamphetamine	Amphetamines, nfd	3103
Amphetamine analogues	Amphetamines, nfd	3104
Amphetamines, nec	Amphetamines, nfd	3199
Cannabinoids (pre‐2015), replaced by 7101	Cannabinoids and related drugs, nfd	3201
Norephedrine	Amphetamines, nfd	3302
Pseudoephedrine	Amphetamines, nfd	3303
Ephedra alkaloids, nec	Amphetamines, nfd	3399
Phenethylamines, nfd	Other Drugs of Concern	3400
MDA	Amphetamines, nfd	3403
MDEA	Amphetamines, nfd	3404
MDMA	Amphetamines, nfd	3405
Phenethylamines, nec	Amphetamines, nfd	3499
Tryptamines, nfd	Hallucinogens	3500
Dimethyltryptamine	Hallucinogens	3503
Lysergic acid diethylamide	Hallucinogens	3504
Psilocybin or psilocin	Hallucinogens	3505
Amyl nitrate	Other drugs of concern	3601
Volatile nitrates, nec	Other drugs of concern	3699
Cathinone	NPS	3701
Methcathinone	NPS	3702
Cathinones, nec	NPS	3799
1‐Benzylpiperazine	NPS	3801
Other stimulants and hallucinogens, nfd	Amphetamines, nfd	3900
Caffeine	Other drugs of concern	3901
Cocaine	Cocaine	3903
Methylphenidate	Amphetamines, nfd	3905
Nicotine	Other drugs of concern	3906
Other stimulants and hallucinogens, nec	Amphetamines, nfd	3999
Anabolic androgenic steroids, nfd	Other drugs of concern	4100
Boldenone	Other drugs of concern	4101
Methandriol	Other drugs of concern	4105
Methenolone	Other drugs of concern	4106
Oxandrolone	Other drugs of concern	4108
Testosterone	Other drugs of concern	4112
Anabolic androgenic steroids, nec	Other drugs of concern	4199
Eformoterol	Other drugs of concern	4201
Fenoterol	Other drugs of concern	4202
Peptide hormones, mimetics and analogues, nfd	Other drugs of concern	4300
Chorionic gonadotrophin	Other drugs of concern	4301
Growth hormone	Other drugs of concern	4304
Other anabolic agents and selected hormones, nec	Other drugs of concern	4999
Antidepressants and antipsychotics, nfd	Other drugs of concern	5000
Monoamine oxidase inhibitors, nfd	Other drugs of concern	5100
Monoamine oxidase inhibitors, nec	Other drugs of concern	5199
Phenothiazines, nec	Other drugs of concern	5299
Fluoxetine	Other drugs of concern	5302
Sertraline	Other drugs of concern	5304
Amitriptyline	Other drugs of concern	5501
Tricyclic antidepressants, nec	Other drugs of concern	5599
Olanzapine	Other drugs of concern	5604
Quetiapine	Other drugs of concern	5605
Other antidepressants and antipsychotics, nfd	Other drugs of concern	5900
Butyrophenones	Other drugs of concern	5901
Lithium	Other drugs of concern	5902
Other antidepressants and antipsychotics, nec	Other drugs of concern	5999
Psychostimulants, nfd	Amphetamines, nfd	0006
Volatile solvents, nfd	Other drugs of concern	6000
Aliphatic hydrocarbons, nfd	Other drugs of concern	6100
Butane	Other drugs of concern	6101
Petroleum	Other drugs of concern	6102
Aliphatic hydrocarbons, nec	Other drugs of concern	6199
Toluene	Other drugs of concern	6201
Aromatic hydrocarbons, nec	Other drugs of concern	6299
Halogenated hydrocarbons, nfd	Other drugs of concern	6300
Halogenated hydrocarbons, nec	Other drugs of concern	6399
Other volatile solvents, nfd	Other drugs of concern	6900
Acetone	Other drugs of concern	6901
Other volatile solvents, nec	Other drugs of concern	6999
Cannabinoids and related drugs, nfd	Cannabinoids and related drugs, nfd	7100
Cannabinoids	Cannabinoids and related drugs, nfd	7101
Cannabinoid agonists	Cannabinoids and related drugs, nfd	7102
Cannabinoids and related drugs, nec	Cannabinoids and related drugs, nfd	7199
Miscellaneous drugs of concern, nfd	Other drugs of concern	9000
Diuretics, nec	Other drugs of concern	9199
Opioid antagonists, nfd	Other drugs of concern	9200
Naloxone	Other drugs of concern	9201
Naltrexone	Other drugs of concern	9202
Opioid antagonists, nec	Other drugs of concern	9299
Other drugs of concern	Other drugs of concern	9999

Abbreviation: NPS, new (and emerging) psychoactive substances.

**TABLE A5:** Ambulance callout description for drug overdose.

CASE_GIVEN_AS
Accident Od/Poison Acid/Alkali
Accident Od/Poison Chng Colour
Accident Od/Poison Unk Status
Accidental Od/Poison Cocaine
Accidental Od/Poison Narcotics
Accidental Od/Poison Not Alert
Accidental Od/Poison Override
Accidental Od/Poison Sob
Accidental Od/Poison Tricyclic
Accidental Od/Poison Uncon
Accidental Overdose
Accidental Poison No Prty Symp
Intention Od/Poison Chng Colou
Intention Od/Poison Unk Status
Intention Poison No Prty Symp
Intentional Od/Poison Acid
Intentional Od/Poison Cocaine
Intentional Od/Poison Narcotic
Intentional Od/Poison Not Aler
Intentional Od/Poison Override
Intentional Od/Poison Sob
Intentional Od/Poison Tricycli
Intentional Od/Poison Uncon
Intentional Overdose
Od Accidental Abnrml Breath
Od Accidental Acid/Alkali
Od Accidental Antidepressants
Od Accidental Cocaine/Deriv
Od Accidental Narcotic/Heroin
Od Accidental No Prty Symp
Od Accidental Severe Sob
Od Accidental Unconscious
Od Accidental Unknown/3rd
Od Accidntl Poison Ctrl Rqust
Od Intentional Abnrml Breath
Od Intentional Acid/Alkali
Od Intentional Antidepressant
Od Intentional Cocaine/Deriv
Od Intentional Narcotc/Heroin
Od Intentional No Prty Symp
Od Intentional Severe Sob
Od Intentional Unconscious
Od Intentional Unknown/3rd
Od Intntnal Poison Ctrl Rqust
Od Violent Or Combative
Od/Poison Abnormal Breath
Od/Poison Acid Or Alkali
Od/Poison Acid/Alkali Violent
Od/Poison Chng Colour Violent
Od/Poison Cocaine/Meth
Od/Poison Cocaine Violent
Od/Poison Narcotics Violent
Od/Poison Not Alert Violent
Od/Poison Override
Od/Poison Sob Violent
Od/Poison Tricyclic Violent
Od/Poison Violent Override
Od/Poison Violent Unk Status
Od/Poison: Antidepressants—Poision Info Cr Req
Od/Poison: Cocaine/Deriv—Poision Info Cr Req
Od/Poison: No Prty Symp
Od/Poison: Not Alert—Poision Info Cr Req
Od/Poison: Poision Info Cr Req
Od/Poison: Unknown/3rd Party—Poision Info Cr Req
Od/Poison: Violent—Poision Info Cr Req
Od/Poisoning Changing Colour
Od/Poisoning Narcotics
Od/Poisoning Not Alert
Od/Poisoning Override
Od/Poisoning Override 1c
Od/Poisoning Unconscious
Od/Poisoning Unknown Status
Overdose
Overdose Accidental Not Alert
Overdose Accidental Override
Overdose Intentional Not Alert
Overdose Intentional Override
Overdose/Poisoning
Overdose: Accidental No Prty Symp
Overdose: Accidental Violent
Overdose: Intentional No Prty Symp
Overdose: Intentional Violent
Overdose/Poisoning
Overdose/Ingestion/Poisonings

**TABLE A6:** Classification of drug and alcohol‐related health events using ICD‐10 codes.

Category	Code
Hospital stays for overdose/poisoning	
Alcohol	X45, X65, Y15, T51.0
Opioids	T40.0–T40.4, T40.6
Cannabis	T40.7
Amphetamines	T43.6
Cocaine	T40.5
Multiple drugs	(> 1 of the above)
Hospital stays for substance use disorders	
Alcohol	F10.x
Opioids	F11.x
Cannabis	F12.x
Sedatives	F13.x
Cocaine	F14.x
Hallucinogens	F16.x
Stimulants	F15.x
Multiple drugs	F19.x
Hospital stays for infections that have been related to injecting drug use	
Skin/soft tissue infections	A48.0, Lo2.x, L03.x, L08.8, L08.9, L97.x, L98.4, L98.8, L98.9, M72.6, R02.x
Endocarditis	B37.6, I33.x–I39.x, T82.6
Osteomyelitis	M46.2, M46.3, M46.4, M86.x, M89.9
Bacteraemia/sepsis	A40.x, A41.x, B37.7, R57.2
CNS infections	G06.0. G06.1, G06.2
Septic arthritis	M00.x
Hospital stays for liver‐related causes	
Viral hepatitis	B15.x–B19.x
Liver cancer	C22.x
Fibrosis and cirrhosis	K74.x
Hospital stay for HIV‐related causes	B20.x–B24.x
Hospital stays due to other causes	
Psychosis	F22.x–F25.x, F28.x–F29.x, F30.2, F31.2, F31.5
Self‐harm	X60.x–X84.x, Y87.0
Depression	F32.0, F32.1, F32.2, F32.8, F32.9, F33.x, F34.1, F38.x, F39.x
Other mental disorders (excl. SUD)	F00.x–F09.x, F16.x–F18.x, F21.x, F26.x, F27.x, F30.1, F30.3–F31.1, F31.6–F31.9, F34.0, F34.2–F41.1, F41.3–F43.1, F43.3–F99.x
Accidental injuries (excl. overdose)	V01.x–X39.x, X50.x–X59.x
Assault	X85.x–Y09.x

**TABLE A7:** Annual incarceration dynamics by gender from 2000 to 2021.

Year	Sex	First incarceration	Incarcerations	Releases	People incarcerated
2000	Female	1518	1518	1288	1518
2000	Male	11,871	11,871	8791	11,871
2001	Female	1273	1839	1834	2080
2001	Male	9065	13,205	12,715	16,362
2002	Female	1027	1811	1817	2140
2002	Male	7285	13,292	13,085	17,462
2003	Female	1329	2278	2301	2641
2003	Male	9186	16,400	16,371	21,342
2004	Female	1358	2483	2497	2897
2004	Male	8817	17,470	17,410	23,007
2005	Female	1384	2607	2678	3076
2005	Male	8699	18,110	18,641	24,344
2006	Female	1420	2751	2769	3215
2006	Male	8488	18,739	19,056	25,086
2007	Female	1469	2878	2955	3406
2007	Male	8215	19,294	19,741	26,017
2008	Female	1546	3006	2949	3562
2008	Male	8310	19,682	19,222	26,883
2009	Female	1484	2940	3102	3611
2009	Male	7997	19,201	20,347	27,218
2010	Female	1375	2879	2983	3494
2010	Male	7363	19,033	19,850	26,707
2011	Female	1299	2822	2966	3406
2011	Male	7016	18,840	19,672	26,318
2012	Female	1287	2984	3051	3477
2012	Male	6271	18,256	18,958	25,490
2013	Female	1280	3061	3139	3592
2013	Male	6293	18,839	19,217	26,059
2014	Female	1186	3032	3081	3568
2014	Male	5779	18,113	18,555	25,793
2015	Female	1390	3416	3389	3979
2015	Male	6722	20,097	19,936	28,157
2016	Female	1606	3866	3908	4519
2016	Male	7058	21,136	21,745	30,329
2017	Female	1604	4036	4159	4774
2017	Male	6622	20,771	21,462	30,339
2018	Female	1569	3991	4153	4751
2018	Male	6634	21,087	21,824	30,882
2019	Female	1624	4151	4260	4852
2019	Male	6998	22,067	22,680	31,858
2020	Female	1431	3841	4047	4573
2020	Male	6171	20,694	22,307	30,866
2021	Female	1239	3419	3585	4089
2021	Male	5378	19,317	20,758	29,063

*Note:* The table presents yearly counts of individuals entering incarceration for the first time (First incarceration), total incarceration events (Incarcerations), releases from incarceration (Releases), and the number of individuals held in custody for any duration during the year (People incarcerated). Data are disaggregated by gender and show consistent disparities in incarceration rates and trends over time.
